# Ocular safety of intravitreal ethylene diamine tetra acetic acid (EDTA): An experimental feasibility study

**DOI:** 10.1016/j.toxrep.2023.04.002

**Published:** 2023-04-06

**Authors:** Amin Zand, Khalil Ghasemi Falavarjani, Mozhgan Rezaei Kanavi, Abbas Habibi, Pasha Anvari, Sayyed Amirpooya Alemzadeh

**Affiliations:** a Eye Research Center, The Five Senses Health Institute, Rassoul Akram Hospital, Iran University of Medical Sciences, Tehran, Islamic Republic of Iran; b Ocular Tissue Engineering Research Center, Shahid Beheshti University of Medical Sciences, Tehran, Islamic Republic of Iran; c Stem Cell and Regenerative Medicine Research Center, Iran University of Medical Sciences, Tehran, Islamic Republic of Iran

**Keywords:** Intravitreal injection, Ethylene diamine tetra acetic acid, Glial fibrillary acidic protein, Rabbit, Retina toxicity, Electroretinography

## Abstract

Ethylene diamine tetra acetic acid (EDTA) is a chelating component that is able to diminish oxidative reactivity and can be a potential neuroprotective drug in various ocular diseases. For assessing the safety of intravitreal EDTA, 10 rabbits were allocated and divided into 5 groups. Right eyes of the animals received intravitreal EDTA (112.5, 225, 450, 900 and 1800 µg /0.1 ml). Fellow eyes were considered as controls. Clinical examinations and electroretinography (ERG) were performed at the baseline and on day 28. The enucleated eyes were subjected to hematoxylin and eosin (H&E) staining, immunohistochemistry for glial fibrillary acidic protein (GFAP) and the terminal deoxynucleotidyl transferase dUTP nick end labeling (TUNEL) test. Clinical examinations, H&E staining and TUNEL assay were unremarkable. The ERG test did not exhibit any significant alteration compared to the baseline values, except for a significant decrease in just one measurement of the eyes injected with 225 µg EDTA. The mean scores of GFAP immune reactivity in the eyes injected with 112.5 and 225 µg EDTA indicated a non-significant reaction. The scores in higher doses were significant. We suggest intravitreal EDTA with a dose threshold of < 450 µg should be studied for ratification of the safe dose.

## Highlights

• Oxidative stress has a key role in inflammatory processes causing retinal, choroidal and optic nerve diseases.
• EDTA is a chelating component that is able to diminish oxidative reactivity and can be a neuroprotective drug.
• This study is performed to identify any observable toxicity sign of intravitreal EDTA, in rabbit eyes.
• Intravitreal EDTA with the dose levels of < 450 µg can be investigated in future studies to confirm the safe dose.

## Graphical Abstract

**Figure:**
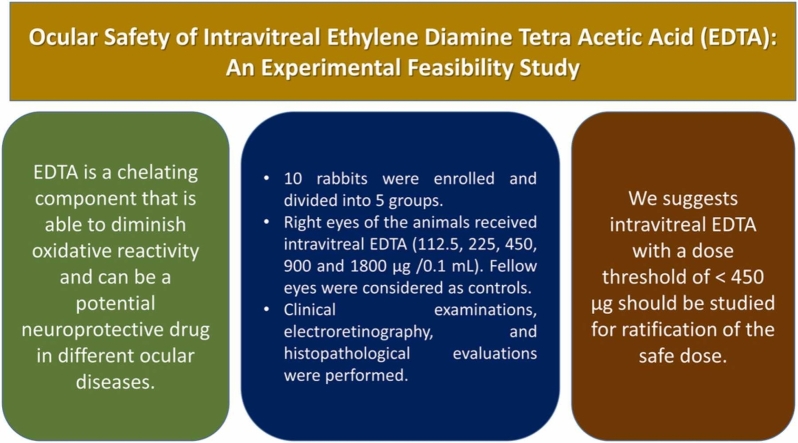

## 1 Introduction

Oxidative stress has a key role in inflammatory processes causing ocular diseases. Previous studies have shown the adverse effect of inflammatory reactions in retinal, choroidal and optic nerve diseases [3], [4], [1], [5], [2]. In diabetic retinopathy, steroids that are potent anti-inflammatory agents play a main role in the treatment of macular edema [6]. Similarly, oxidative stress plays a role in instigating and progressing of dry and wet age related macular degeneration (AMD) [1]. Although intravitreal injections of anti-vascular endothelial growth factors (VEGF) are the standard treatment in choroidal neovascularization, the response to the treatment is not satisfactory in many cases, possibly because the inflammatory pathways are not taken into consideration [7]. In glaucoma, in half of the patients receiving proper treatment with intraocular pressure (IOP)-reducing agents, optic nerve degeneration persists [8]. In non-arteritic anterior ischemic optic neuropathy (NAION), the cellular degeneration may continue after an acute event, possibly because of inflammatory reaction and oxidative stress [5]. Considering the main role of oxidative stress in the progression of these diseases, the use of antioxidants, has received growing attention [10], [9].

Heavy metals such as lead are neurotoxic agents that affect neural organs including the eye. These metals have a melanin affinity and their concentration is highest in melanin-rich components of the eye such as pigment epithelium and choroid. They compete with other metals including copper and zinc for binding to the same sites in melanosomes, especially in the retinal pigmented epithelium cells [11]. These heavy metals can play a role in the pathogenesis and progression of some ocular diseases. There may be an association between heavy metals exposure and incidence of glaucoma in urban areas [12]. Hwang et al. [13] reported that an increased level of blood lead was associated with AMD progression. Elmorsy and Parrey [14] suggested a high blood lead level maybe a contributing factor in diabetic retinopathy.

Chelator agents are antioxidant drugs that are routinely used in medical practice especially for heavy metal (e.g. lead) poisoning [15]. These products are also used for risk reduction in patients with neurodegenerative and cardiovascular disorders [17], [16]. These agents are chemicals that bind to and remove ions from solutions. Ethylene diamine tetra acetic acid (EDTA) is a chelating component that is able to "sequester" metal ions (e.g. Pb^2+^, Fe^3+^ or Ca^2+^). After EDTA is bound to these metal ions, they exhibit diminished oxidative reactivity [18]. Chelation can reduce the formation of reactive oxygen species and thus, diminish inflammation and apoptosis [8]. Roussel et al. [19] showed that plasma peroxide levels (monitored by malon dialdehyde) and DNA damage (monitored by formamido pyrimidine-DNA glycosylase sensitive sites) decreased by 20 % and 22 %, respectively 5 weeks after intravenous administration of an EDTA chelation cocktail. Hence, in addition to being a heavy metal chelator, this agent has an antioxidant role.

In ophthalmology, topical edetate disodium (disodium salt of EDTA) is used in the management of band keratopathy [20]. Topical administration of this agent has been shown to penetrate the ocular surface. The addition of EDTA promotes the corneal penetration of topical ocular drugs [21]. Experimental studies have shown that topical EDTA significantly reduces lens opacification, improves surveillance of ganglion cells and diminishes demyelinization of glaucomatous optic nerve in animal models [8], [22]. Furthermore, it was used as an added component (with 0.5 mg/ml concertation) to intravitreal vancomycin, gentamicin, and dexamethasone [23].

Chemical features of this agent make it suitable for intraocular administration. It is metabolically inert and does not accumulate in the eye [24]. It is water soluble with a pH of 8.0, and is therefore in the safe range for intraocular tissues. Similarly, the osmolarity of the diluted EDTA is within the safe range for intravitreal injections (270–330 mOsmol/L) [18], [25]. EDTA is an anionic hydrophilic agent, and the main rout for its clearance is through aqueous humor outflow [26]. Therefore, according to the literature and chemical features of EDTA, intravitreal injection of this agent (as a heavy metal chelator and antioxidant) may prove useful in ocular neuroprotection. Potentially, this component (as a heavy metal chelator) may be useful in reducing inflammatory and degenerative processes of various disorders of the retina and optic nerve including AMD, diabetic retinopathy, some optic neuropathies, and their relevant conditions, as previous studies have revealed the role of heavy metals, including lead, in deterioration of some retinal disorders [13], [14]. Furthermore, EDTA has antioxidant effects, and the role of these types of components in protecting the retina from ischemic injuries is well known [27], [10], [9]. The safety of intravitreal injection of EDTA has not been evaluated until now. The aim of this feasibility study was to identify any possible toxicity signs for different concentrations (112.5–1800 µg/0.1 ml) of intravitreal EDTA in rabbit eyes, and provide a baseline for setting dose levels in future studies with larger sample sizes.

## 2 Methods

### 2.1 Animals

Ten New Zealand male albino rabbits (2–3 kg) were used in this research. The animals were divided into the five groups (2 animals in each group) for evaluating the ocular safety of five different doses of intravitreal EDTA injection. The animals were maintained in accordance with the principles outlined in the Guide for the Care and Use of Laboratory Animals, Eighth Edition, National Academy Press, Washington DC, 2010 and were cared in agreement with the statement for the use of animals based on the Association for Research in Vision and Ophthalmology guidelines. The Ethics Committee of the Iran University of Medical Sciences approved the protocols of the study ( IR.IUMS.FMD.REC 1396.9511257010). Slit-lamp biomicroscopy and indirect ophthalmoscopy were done at the baseline, and animals with corneal, lens or retinal disorders were excluded. The animals were anesthetized before all procedures using a mixture of 10 % ketamine hydrochloride (50 mg/kg, Alfamine; Alfasan, Woerden, Netherland) and 2 % xylazine hydrochloride (5 mg/kg, Rompun; Bayer, Leverkusen, Germany). Topical anesthesia was applied using tetracaine eye drops (Anestocaine, Sina Darou Laboratories, Tehran, Iran). The pupil was dilated using topical tropicamide 0.5 % (Sina Darou Laboratories, Tehran, Iran).

### 2.2 Intravitreal injections

EDTA was obtained from a commercially available Edetate disodium 0.05 % (50 mg/ml, 10 ml, pH: 8.0 and osmolarity of 1500 mOsmol/L, SERB company, France). 5 % dextrose water (DW5 %, recommended ingredient for diluting of EDTA) was used to provide different concentrations of 112.5, 225, 450, 900, and 1800 µg of the drug in 0.1 ml [28]. The dosage of 450 µg of EDTA was considered the mid-dose for intraocular injections based on a previous study that showed the efficacy of topical one drop administration of 0.86 % (≈ 450 µg) EDTA in the conjunctival sac, by ameliorating ocular oxidative stress and neurodegenerative processes in rats with induced elevated IOP [8]. One of the stated EDTA doses was injected in the right eye of each rabbit in each group. To assess the confounding effect of the intravitreal injection procedure itself on the results of histopathological and immunohistochemical evaluations, the fellow eyes of animals were designed as either Sham (no injection) or DW5 % injection. In each group, an equal volume of the DW5 % was injected in the fellow eye of each rabbit. No intravitreal injection was performed in the fellow eye of the other rabbit in that group. A blinded skilled ophthalmologist performed all procedures in a sterilized environment. After applying periocular and conjunctival betadine 5 %, and reducing intraocular pressure by withdrawing 0.1 ml of aqueous fluid, intravitreal injections were performed by a 30-gauge needle in the supero-temporal quadrant, 1.5 mm behind the limbus. The procedure was executed carefully to minimize possible traumatization of retina as even focal injury might affect the results of histopathological and immunohistochemical evaluations, and causing a confounding effect [29], [30].

### 2.3 Clinical examinations

Ocular examinations were performed by a portable slit-lamp microscope (for presence or absence of ocular surface irritancy and anterior chamber reaction) and indirect ophthalmoscope in order to evaluate any disorders (for presence and severity of vitreous haziness and chorioretinal ischemia/necrosis) on the first day following the intravitreal injection and subsequently on days 3, 7, 14 and 28. The grading system of vitreous haziness and chorioretinal ischemia/necrosis is described in Table 1 [31].

**Table 1:** Grading system of clinical findings.

Clinical finding	Grade	Description
Vitreous haziness	0	None, no clinical findings
	1	Minimal, posterior pole clearly visible
	2	Mild, posterior pole details slightly hazy
	3	Moderate, posterior pole details very hazy
	4	Marked, posterior pole details barely visible
	5	Severe, fundus details not visible
Chorioretinal ischemia/necrosis	0	None, no clinical findings
	1	One quadrant involvement
	2	Two quadrants involvement
	3	>Two quadrants involvement

### 2.4 Electrophysiological testing

Electrophysiological examinations were performed under general anesthesia at the baseline before intravitreal injection and on day 28 before enucleation procedure. Full field electroretinography (ERG) responses were recorded by a mini-Ganzfeld bowel and a Roland electroretinography system (Ronald Consult, Wiesbaden, Germany). Pupil dilation was achieved by the application of topical tropicamide 0.5 % (Sina Darou Laboratories, Tehran, Iran)*. ERG-*jet contact lens (each having a gold ring) electrodes (ERG-Jet, Fabrinal Company, Switzerland) served as positive electrodes. The electrodes were coated with a drop of 2 % methylcellulose gel (EyeGel, Eyeol, England) before being placed over the corneal surface. The ground and negative needle electrodes (Red Dot electrode, 2282, 3 M Company, Canada) were placed in the subcutaneous tissue of the forehead and bi-temporal areas of the rabbit head, respectively. Dark adaption was scheduled for 20 min. Then, scotopic responses (rod and maximal combined) were recorded by the use of scotopic flash ERG. Subsequently, after 10 min of light adaptation, a photopic flash ERG, documented photopic (cone) responses. This procedure followed the protocol of the International Society for Clinical Electrophysiology of Vision (ISCEV) standards [32]. The mean of ERG responses for each group (including Sham, DW5 %, and different doses of EDTA injected eyes) were calculated. Changes were considered significant if the post injection a- or b-wave amplitudes declined by ≥ 66 % of the baseline values [33].

### 2.5 Histopathology, immunohistochemistry, and apoptotic evaluation

Twenty-eight days after the injections, the animals were sacrificed by an intravenous injection of ketamine (500 mg/kg) under general anesthesia. After enucleation of the eyes and fixation in 10 % formalin, tissues were processed and embedded into paraffin. The sections were prepared with 5 µm thickness and in 3 different horizontal planes (200 µm apart) which included the optic nerve. Then, they were stained with hematoxylin and eosin (H&E). A blinded ocular pathologist assessed them, via light microscopy, for any evidence of inflammation, hemorrhage, or atrophy. To evaluate changes of retinal glial cells (including Müller cells) immune reactivity, glial fibrillary acidic protein (GFAP) (Z 0334; Dako, Glostrup, Denmark) staining was used. The scoring of GFAP results were described from 0 to 5. Briefly, 0 was the score given when there was no staining and 5 was the score given when there was a diffuse staining of glial cells involving the full length of the cells [34]. In each group, a mean score of ≥ 2.5 was considered to be significant [35].

The apoptosis was evaluated with the terminal deoxynucleotidyl transferase dUTP nick end labeling (TUNEL) kit (In Situ Cell Death Detection Kit, Fluorescein; 11684795910, Roche Company, Germany) by detecting apoptotic cells’ DNA segments in the retina. Briefly, after preparation of the slides according to the manufacturer’s guidelines, each slide (except negative controls) was treated with a mixture of enzyme and label solutions as the solution of TUNEL reaction. Negative controls were treated with merely label solution. After dark incubation, the slides were rinsed with phosphate-buffered saline, and then were evaluated and imaged with an inverted fluorescence microscope (Olympus IX71; Tokyo, Japan) equipped with a digital camera (Olympus U-TV0.63XC; Tokyo, Japan) to capture the images of interest. Any evidence of apoptosis in the EDTA-injected cases was compared with those in the controls.

## 3 Results

### 3.1 Clinical examinations

Post-injection slit lamp biomicroscopy and indirect ophthalmoscopy were performed on days 1, 3, 7, 14 and 28. During the follow up visits, no signs of anterior chamber reaction, vitreous haziness, or chorioretinal lesions were detected in any eyes.

### 3.2 Electrophysiological findings

No significant reduction in a- or b-wave mean amplitudes of any responses were observed in any groups, except for a significant reduction in a-wave mean amplitude of maximal combined rod-cone response in the 225 µg EDTA injected eyes [Table 2, Table 3, Table 4].

**Table 2:** The mean changes in the amplitude of rod response of full-field electroretinography recording after intravitreal injection of ethylene diamine tetra acetic acid (EDTA).

	Rod Response			
Group	a-wave (micro Volt)		b-wave (micro Volt)	
	Difference	Difference (%)	Difference	Difference (%)
Sham	-0.86	68.47	-0.92	12.33
DW5 %^a^	0.06	13.74	13.66	23.99
112.5 µg EDTA	6.60	30.34	-4.60	46.39
225 µg EDTA	-11.30	-53.82	-17.00	-6.30
450 µg EDTA	-1.70	-19.92	64.10	101.25
900 µg EDTA	3.25	26.48	-15.70	-13.51
1800 µg EDTA	-4.65	-28.10	31.15	47.68

a Dextrose Water 5 %.

**Table 3:** The mean changes in the amplitude of maximal combined response of full-field electroretinography recording after intravitreal injection of ethylene diamine tetra acetic acid (EDTA).

	Combined Rod-Cone Response			
Group	a-wave (micro Volt)		b-wave (micro Volt)	
	Difference	Difference (%)	Difference	Difference (%)
Sham	-10.66	-34.99	3.48	12.51
DW5 %^a^	-0.74	4.99	25.64	32.92
112.5 µg EDTA	2.85	16.00	1.45	48.43
225 µg EDTA	-30.10	-66.97 ^b^	-43.75	-26.53
450 µg EDTA	31.75	221.13	24.8	37.70
900 µg EDTA	-26.15	-57.64	14.05	10.95
1800 µg EDTA	0.10	1.68	39.35	43.04

^a^ Dextrose Water 5 %.

^b^ Significant decline compared to the baseline: ≥ 66 %.

**Table 4:** The mean changes in the amplitude of cone response of full-field electroretinography (ERG) recording after intravitreal injection of ethylene diamine tetra acetic acid (EDTA).

	Cone Response			
Group	a-wave (micro Volt)		b-wave (micro Volt)	
	Difference	Difference (%)	Difference	Difference (%)
Sham	-0.03	27.55	-18.08	-23.03
DW5 %^a^	4.16	118.25	-14.74	11.30
112.5 µg EDTA	-2.45	-23.10	-40.40	-46.23
225 µg EDTA	2.75	52.70	-15.05	-12.71
450 µg EDTA	4.65	91.96	41.85	192.50
900 µg EDTA	11.65	185.09	38.70	46.39
1800 µg EDTA	-2.95	-43.13	-7.05	-7.54

a Dextrose Water 5 %.

### 3.3 Routine histopathology, TUNEL evaluation and GFAP staining

**Fig. 1:**
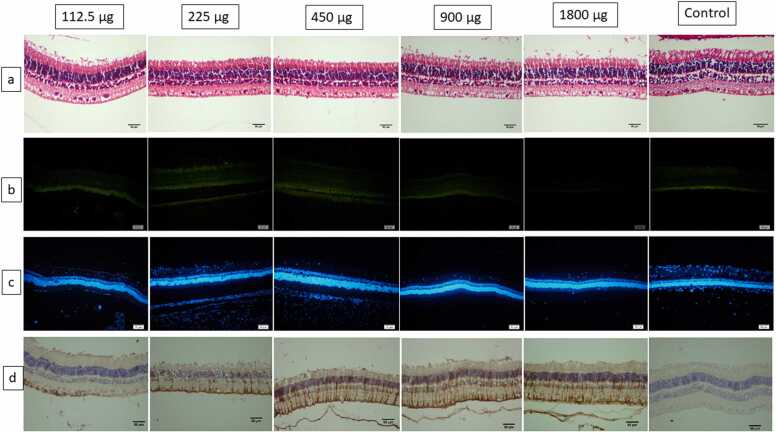
Representative photomicrographs of the retinas after intravitreal injections of 112.5, 225, 450, 900 and 1800 μg in comparison to a control eye. a: Neurosensory retinal layers were unremarkable in the hematoxylin/eosin (H&E) staining. b: Note the lack of apoptotic fluorescent cells in the retinas on terminal deoxynucleotidyl transferase dUTP nick end labeling (TUNEL) assay in all the study groups. c: Corresponding diamidino-phenylindole (DAPI)-stained nuclei (blue). d: A significant immune reactivity for glial fibrillary acidic protein (GFAP) is observed in the 450, 900 and 1800 μg /0.1 ml EDTA injected eyes. Note non-significant immune reactivity of the rabbits’ retinas for GFAP in the 112.5 and 225 μg /0.1 ml injected eyes as well as in the control.

Inflammation, retinal hemorrhage or atrophy were not detected in the H&E-stained sections. In the sections, the retina was artifactually detached from retinal pigmented epithelium, but, the integrity of the retinal layers was preserved. Other ocular structures including lens and cornea did not have any remarkable findings [Fig. 1a].

TUNEL evaluation disclosed no signs of apoptosis in the intravitreal EDTA injected eyes as compared to the control eyes [Fig. 1b-c]. The mean scores of GFAP reactivity in eyes injected with Sham, 112.5 and 225 µg EDTA were 2.0, 2.0, and 1.5, respectively, indicating a non-significant reaction. The mean score for DW5 %, 450, 900 and 1800 µg EDTA injected eyes were 2.8, 3.5, 5.0, and 5.0, respectively, which demonstrated a significant reaction [Fig. 1d].

## 4 Discussion

In the present study, clinical examinations, H&E staining and TUNEL assay did not reveal any signs of toxicity or inflammation in ocular structures for all doses of intravitreal EDTA. In GFAP test, the mean score was lower than the recommended nontoxic score (< 2.5) in the Sham group, and also in 112.5 and 225 µg injected eyes (≤ 2.0), in contrast to the eyes having ≥ 450 µg injections with the toxic scores (≥ 3.5). The mean score was higher in eyes injected with DW5 % in comparison to the Sham group (2.8 vs. 2.0). This difference can be explained by the retinal glial cell activation as a consequence of possible neural cell traumatization and damage during intravitreal injection, which can increase GFAP expression even in response to the focal injury caused by the injection procedure [29], [30]. Although the mean GFAP score of DW5 % injected eyes was slightly higher than the nontoxic score, nevertheless, in both Sham and DW5 % injection groups, other evaluations including clinical examinations, ERG responses, histopathology and TUNEL assay did not exhibit any significant alterations. Therefore, the confounding effect of intravitreal injection procedure itself was limited.

In the EDTA-injected eyes, a significant decline in just one of mean ERG responses was detected in 225 µg EDTA dose, while higher doses had non-significant variations. Previous studies have shown similar discrepancies in ERG recordings in rabbit eyes [36], [37]. One of the plausible explanations for these discrepancies is a high intersession variability in ERG recordings, especially in low sample sizes [33], [38]. Therefore, the ERG findings must be interpreted with caution in experimental studies.

Among groups with higher doses (≥ 450 µg) of EDTA injection, ERG responses, routine histopathology, and TUNEL assay showed no significant abnormal alterations, unlike the GFAP results. This disparity may indicate that higher doses of intravitreal EDTA were sufficient for the induction of retinal glial cells (including Müller cells) dysfunction but their toxic effects were not severe enough to cause retinal atrophic changes, photoreceptors dysfunction or ganglion cells apoptosis. But, this interpretation should be considered with caution due to high intersession variability in ERG recordings, and also low sample sizes in each group of our study.

The safety of intravitreal injection of EDTA has not been evaluated until now. Few studies on the efficacy of topical EDTA as a neuroprotective agent for the treatment of ocular disorders exist [8], [22]. Since some metal ions produce reactive oxygen components, chelation therapy is one of the potential treatment options for cataracts. Zhang et al. [22] showed that the topical administration of a mixture of EDTA and the permeability augmenter methyl sulfonyl methane (MSM) reduced protein-4-hydroxynonenal (HNE) accumulation. This component is a target in inducing oxidative stress and subsequently, lens opacification. EDTA-MSM significantly diminished lens opacifications, lens cells swelling and proliferation and the amounts of protein-HNE components. The investigators documented these findings both in vitro and in vivo in the early phases of cataract development. They showed that the most effective dose was one drop administration of 0.25 % EDTA in the conjunctival sac, but the 0.5 % dose was less effective than the 0.25 %. They did not report any adverse effect of topical EDTA administration in ocular tissues for all tested doses (0.1 %, 0.25 % and 0.5 %) in their clinical or histopathological examinations. In another study by Liu et al. [8] the investigators administrated the topical ocular combination of EDTA-MSM to evaluate its neuroprotective effects in the rat retinal ganglion cells and optic nerve affected by high IOP-induced oxidative stress. They administrated one drop of 0.86 % EDTA in the conjunctival sac of the treated eyes. They demonstrated a reduction in the production of protein-lipid aldehyde and cyclooxygenase-2 (COX-2) enzyme that ameliorated oxidative injury and inflammatory processes in comparison to the controls, respectively. Also, they demonstrated that treatment with EDTA-MSM improved retinal ganglion cells surveillance and reduced demyelinization of optic nerve. Similar to previous studies, the investigators did not find any pathologic disorders in the treated eyes in their clinical and histopathological investigations. Xiao et al.,[39] investigated the effect of EDTA on endotoxin-induced uveitis in rats. Animals were topically treated with EDTA-MSM. The investigators evaluated the number of infiltrating cells, protein, tumor necrosis factor alpha (TNF-α) and prostaglandin E2 (PGE2) in the aqueous humor. The levels of these inflammatory markers were significantly lower in the rats treated with EDTA-MSM in comparison to the controls. Therefore, chelation therapy with EDTA may be useful in the treatment of uveitis. Although the investigators of these few studies did not report any adverse effect of topical EDTA in ocular tissues, it is not sufficient for reaching a conclusion about the ocular safety of the drug. The main goal of the aforementioned studies was the evaluation of topical EDTA efficacy as a potential neuroprotective or anti-inflammatory agent in some particular ocular diseases, and not the evaluation of the safety of the drug. In addition, many ocular drugs are safe when applied topically, but completely toxic for ocular tissues when administrated via intraocular routs. High levels of heavy metals including lead may cause progression of some retinal disorders including AMD and diabetic retinopathy, and EDTA as a heavy metals chelator can potentially retard the progression of these diseases [13], [14]. Furthermore, EDTA as an anti-oxidant component may protect the retina from ischemic injuries [27], [10], [9].

Finding the safe dose of intravitreal EDTA, as investigated in our study, would be of great value because intravitreal injection of EDTA in comparison with its topical administration provides higher intraocular concentrations that may be associated with better drug efficiency.

This study had some limitations. The first and main limitation was the small sample size that restricts the interpretation of our findings. But, this was a feasibility study with the main purpose of finding observable signs of the drug toxicity, and finding a baseline for setting more proper dose levels in future experimental studies with larger sample sizes. In addition, high costs (including costs for animals housing, performing ERG and histopathological and immunohistochemical evaluations) restricted the number of subjects that could be recruited in our study. Second, a single ERG measurement at 28 days may fail to detect transient short-term alterations in the retinal function. However, these results can provide a starting point for the promotion of intravitreal EDTA-based future investigations.

There are several guidelines for testing chemicals on animals. One of these guidelines for designing experimental studies on the eyes is the protocol of the Organization for Economic Cooperation and Development (OECD, No. 405), which suggests a single dose of a testing substance on one eye of each animal, with the untreated fellow eye being considered as a control [40]. According to this guideline, the initial test should be used on one animal, and if an irritant or negative response is found, a confirmatory test should be applied on two additional animals. Based on this guideline and the results of our feasibility study, we suggest that subsequent studies should apply intravitreal EDTA at each dose of 225 and 112.5 µg/0.1 ml on one eye of at least two animals (with untreated fellow eyes as controls) in order to determine and confirm the safe dose threshold of the drug.

## 5 Conclusion

This study suggests that intravitreal EDTA with the dose levels of < 450 µg can be explored in future experimental studies for confirmation of the safe dosage of the drug and for assessing its potential intraocular antioxidative and neuroprotective effects. Therefore, further studies with larger sample sizes and longer follow-up periods are required.

## Funding information

This work was supported by a grant numbered IUMS-97-04-75-12362 of the Iran University of Medical Sciences, Tehran, Iran.

## CRediT authorship contribution statement

**Khalil Ghasemi Falavarjani** : Conceptualization,Funding acquisition, Investigation, Project administration, Resources, Writing – review & editing. **Amin Zand**: Conceptualization, Formal analysis, Investigation, Methodology, Project administration, Writing – review & editing. **Mozhgan Rezaei Kanavi:** Formal analysis, Investigation, Methodology, Writing – review & editing. **Pasha Anvari:** Methodology, Project administration. **Abbas Habibi:** Methodology, Project administration, Resources. **Sayyed Amirpooya Alemzadeh:** Project administration, Resources.

## Declaration of Competing Interest

The authors declare that they have no known competing financial interests or personal relationships that could have appeared to influence the work reported in this paper.

## Data Availability

Data will be made available on request.
